# RADseq analyses reveal concordant Indian Ocean biogeographic and phylogeographic boundaries in the reef fish *Dascyllus trimaculatus*

**DOI:** 10.1098/rsos.172413

**Published:** 2019-05-29

**Authors:** E. M. Salas, G. Bernardi, M. L. Berumen, M. R. Gaither, L. A. Rocha

**Affiliations:** 1 Section of Ichthyology, California Academy of Sciences, 55 Music Concourse Drive, San Francisco, CA 94118, USA; 2 Department of Ecology and Evolutionary Biology, University of California Santa Cruz, 100 Shaffer Rd, Santa Cruz, CA 95060, USA; 3 Red Sea Research Center, Biological and Environmental Science and Engineering Division, King Abdullah University of Science and Technology, Thuwal, Saudi Arabia; 4 Department of Biology, Genomics and Bioinformatics Cluster, University of Central Florida, 4110 Libra Drive, Orlando, FL 32816, USA

**Keywords:** population genomics, outlier loci, connectivity, Indian Ocean, Red Sea, *Dascyllus trimaculatus*

## Abstract

Population genetic analysis is an important tool for estimating the degree of evolutionary connectivity in marine organisms. Here, we investigate the population structure of the three-spot damselfish *Dascyllus trimaculatus* in the Red Sea, Arabian Sea and Western Indian Ocean, using 1174 single nucleotide polymorphisms (SNPs). Neutral loci revealed a signature of weak genetic differentiation between the Northwestern (Red Sea and Arabian Sea) and Western Indian Ocean biogeographic provinces. Loci potentially under selection (outlier loci) revealed a similar pattern but with a much stronger signal of genetic structure between regions. The Oman population appears to be genetically distinct from all other populations included in the analysis. While we could not clearly identify the mechanisms driving these patterns (isolation, adaptation or both), the datasets indicate that population-level divergences are largely concordant with biogeographic boundaries based on species composition. Our data can be used along with genetic connectivity of other species to identify the common genetic breaks that need to be considered for the conservation of biodiversity and evolutionary processes in the poorly studied Western Indian Ocean region.

## 1. Introduction

Coral reefs are home to a diversity of fish species that originated despite the presence of few geographical barriers to larval dispersal [1]. To understand the origins of marine biodiversity, evolutionary biologists have often turned to the highly diverse Indo-Pacific realm (as defined by Kulbicki *et al.* [2]), a large biogeographic region spanning from the Red Sea to the Central Pacific. This realm includes many wide-ranging species that, upon close study, often show structured populations [3], revealing the evolutionary and historical mechanisms that operate in the oceans. These studies can also be relevant to marine resource management and conservation planning. For example, in Hawaii, assessing the connectivity of multiple species enabled the identification of common genetic breaks that can be used to delineate management units [4].

**Figure 1.**
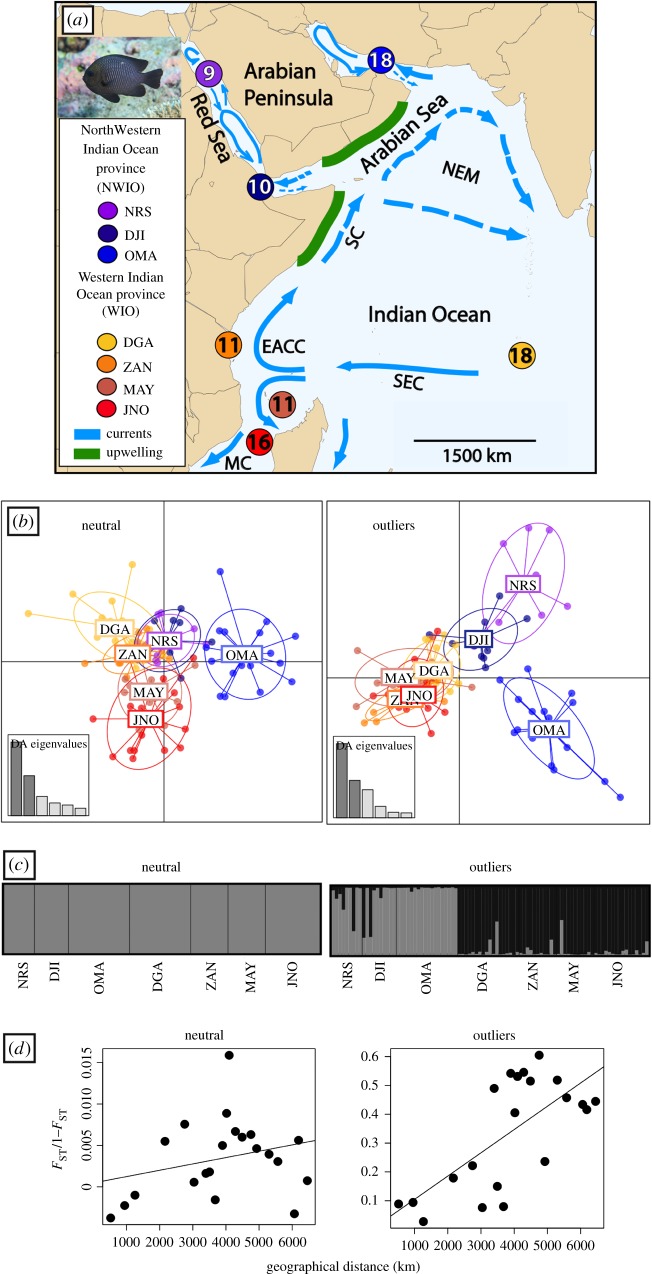
(*a*) Study region with populations and sample sizes (circles) indicated (NRS, Northern Red Sea; DJI, Djibouti; OMA, Oman; DGA, Diego de García, Chagos; ZAN, Zanzibar; MAY, Mayotte; JNO, Juan de Nova, Scattered Islands). Summer upwelling and currents are shown; dashed lines indicate winter reversals. Currents (C): NEM, North East Monsoon C; SC, Somali C; EACC, East African Coastal C; MC, Mozambique C; SEC, South Equatorial C. (*b*) DAPC for neutral (*n* = 1117) and outlier loci (*n* = 25). (*c*) structure plot with most likely *K* for neutral (*K* = 1) and outlier loci (*K* = 2). (d) Isolation by distance (IBD) for neutral loci, mantel test *p* = 0.1990, *r*^2^ = 0.0756, *y* = 8 × 10^−7^*x* + 0.0005; and outlier loci, *p* = 0.016, *r*^2^ = 0.4801, *y* = 8 × 10^−5^*x* + 0.0239. *Dascyllus trimaculatus* picture by Tane Sinclair-Taylor.

Over the past two decades, there have been numerous phylogeographic studies of Indo-Pacific reef fishes, but comparatively few have examined patterns of population connectivity within the Western Indian Ocean [5,6]. At the largest scale, and based on species distributions, this biogeographic region was divided into the Northwestern Indian Ocean (NWIO) and the Western Indian Ocean (WIO) provinces ([2], figure 1). In general, biogeographic boundaries are created by one of two mechanisms [7]: either the boundary is where two separate faunas meet, or the boundary itself is the engine of faunal separation. In the latter case, sister species are expected to occur across the boundary, and in populations that have not yet reached the stage of speciation, the biogeographic boundary coincides with a population separation—a phylogeographic break. The goal of this study was to assess phylogeographic patterns in *Dascyllus trimaculatus* and determine whether they matched established biogeographic boundaries.

In general, studies that have sampled populations in the Indian Ocean find results ranging from no genetic structure [5,8–15], to little differentiation [16–18]. However, some of these studies have limited geographical coverage. In some cases, strong genetic breaks between the Red Sea (a hotspot of endemism) and the Indian Ocean were observed [19,20]. Biogeographic studies on the distribution and evolutionary origin of endemic species show changes in species composition between the Red Sea, Gulf of Aden and Arabian Sea [21,22]. Within the WIO province, there appears to be genetic breaks between the Seychelles and populations on the African coast in some species, such as the parrotfish *Scarus ghobban* [18] and the mangrove crab *Neosarmatium meinerti* [23]. Some datasets also show evidence of a population genetic separation between the Chagos Archipelago and the rest of the WIO, for example in the butterflyfish *Chaetodon trifasciatus* [24]. Detailed studies of the widespread fish species *Epinephelus merra* and *Myripristis berndti* also showed evidence of structure within the WIO [25,26].

The three-spot damselfish, *D. trimaculatus* is an abundant species found throughout the Indo-Pacific [27], from the Red Sea to French Polynesia, and has many characteristics that are ‘typical' of a coral reef dwelling damselfish. It spawns 2–3 times a month for several successive months [28] producing demersal eggs that are guarded for 2–3 days [29], and has a pelagic larval stage that lasts 22–26 days [30]. Juveniles generally settle on anemones, which they abandon once they are large enough to avoid predation, to find shelter in reef crevices nearby [27,29]. *Dascyllus trimaculatus* belongs to a species complex that comprises four species, *D. trimaculatus, D. albisella, D. strasburgi* and *D. auripinnis* [31]. The recent divergence of these species is consistent with a parapatric speciation scenario [31], as *D. trimaculatus* occupies nearly the entire Indo-Pacific range except the Hawaiian Archipelago, the Marquesas Islands, and the Line Islands, whereas the closely related *D. albisella, D. strasburgi* and *D. auripinnis* are only present in those restricted peripheral ranges, respectively [31]. *Dascyllus trimaculatus* populations show patterns of allopatric divergence—as the Pacific Ocean populations are distinct from those of the Indian Ocean, these populations were probably isolated by restricted water flow in the Sunda Shelf during the Pleistocene sea-level changes [31]. In the Pacific Ocean, where all four species of the complex are present, genetic studies have found a lack of congruence between colour morphs and genetic groups. For example, *D. auripinnis,* which has a bright yellow ventral surface and fins, is not fully genetically differentiated from *D. trimaculatus*. By contrast, only *D. trimaculatus* is present in the Indian Ocean and mitochondrial DNA (d-loop) and microsatellite comparisons showed these populations belong to a single clade [31].

Here, we use single nucleotide polymorphisms (SNPs) developed using restriction-site associated DNA sequencing (RADseq) to assess the population structure in Indian Ocean *D. trimaculatus*. Since recent biogeographic studies have found that in some species genetic boundaries match biogeographic regions in the Indian Ocean [32], and because *D. trimaculatus* shows moderate amounts of genetic structure throughout its range with evidence of divergence in the species complex in peripheral habitats [31], we considered the possibility of finding genetic divergence in peripheral areas where biogeographic boundaries have been proposed (such as the Red Sea). Not only did sea-level changes repeatedly isolate the Red Sea from the Indian Ocean during the Pleistocene [19], but currently, the presence of an upwelling along the Horn of Africa and the coast of Oman could further isolate Red Sea and Arabian Peninsula populations from those of the Indian Ocean [21]. In the Red Sea, the pelagic larval duration (PLD) of *D. trimaculatus, D. aruanus* and *D. marginatus* decreases as sea surface temperatures and food availability increase from north to south [33]. These results suggest that local environment variation affects the life history of *D. trimaculatus*, which in turn might influence its genetic structure. Currents near the African coast may also affect the genetic connectivity of *D. trimaculatus* in the Indian Ocean. The main oceanic current in the Indian Ocean is the South Equatorial Current (SEC, figure 1, [18]). This current splits near Tanzania into two continental currents, the East African Coastal Current (EACC) that flows north towards Somalia, and the Mozambique current that flows south and produces eddies in the Mozambique Channel [15,18], potentially isolating populations located in the Mozambique Channel from populations further north along the African coast.

In this study, we assessed differences between populations from the Red Sea, Arabian Peninsula, African coast, Mozambique Channel and Chagos Archipelago. We also tested for population divergence between the biogeographic NWIO and WIO provinces. To the best of our knowledge, this is one of the first studies based on extensive genomic data to compare population genetic structures in the Western Indian Ocean that simultaneously covers these two provinces.

## 2. Material and methods

### 2.1. Sample collection

A total of 93 individuals from seven populations were collected while diving or snorkelling between 1998 and 2013 (figure 1). Populations were grouped, following Kulbicki *et al.* [2], into Northwestern Indian Ocean Province, NWIO (Northern Red Sea: NRS, Djibouti: DJI and Oman: OMA); and Western Indian Ocean Province, WIO (Diego Garcia, Chagos Archipelago: DGA, Zanzibar, Tanzania: ZAN, Mayotte, Comoros Archipelago: MAY and Juan de Nova, Scattered Islands, Mozambique Channel: JNO, figure 1). The NRS population consisted of six individuals from Eilat, Israel and three from Jeddah, Saudi Arabia. Some of the samples were part of the earlier studies including individuals from Eilat, Oman, Mayotte and Zanzibar [31,34].

### 2.2. RADseq library preparation and sequencing

Genomic DNA was extracted using the Qiagen DNeasy animal blood and tissue kit (Qiagen, Valencia, USA). The library was prepared using the double-digest RADseq protocol [35], with modifications (see electronic supplementary material, methods) and sequenced on a single Illumina HiSeq 2000 lane, at the UCLA Neuroscience Genomics Core facility. Raw data were de-multiplexed, quality filtered and trimmed to 95 bp, using the ‘process_rad_tags' script available in stacks v. 1.09 [36]. Loci were assembled using the stacks ‘de novo_map.pl' pipeline, while the ‘populations' script was used to filter loci and create output files (for raw data filtering and loci assembly see electronic supplementary material, methods). Loci were shared between the seven populations (*p* = 7), in at least 65% of individuals within a group (*r* = 0.65) and with a coverage of 8× (*m* = 8). We used only the first SNP of each sequence and removed loci with minor allele frequencies lower than 1.5% (i.e. at least two individuals must have the unique allele). Our quality control and filtering resulted in a total of 1174 loci and a data matrix that was 84% complete. We used pgdspider 2.0 [37] to convert the resulting structure files into other formats.

### 2.3. Data analysis

First, we conducted population genetic analysis with all the loci (*n* = 1174). Then, to gain more perspective on the patterns of genetic structure and its potential mechanisms, we separated neutral from outlier loci, and repeated the population genetic analyses with the neutral subset and the subset of outliers that had higher than expected FST based on neutral expectations (see below).

To identify outlier loci, we used three methods. First, we used the modified fdist approach [38] implemented in arlequin [39], which uses a hierarchical island model and simulates an *f*_ST_ null distribution across loci as a function of heterozygosity and determines outliers as being those outside of the distribution using a 99% confidence interval. We ran 50 000 simulations with 100 demes per group, with minimum and maximum expected heterozygosities of 0 and 0.5, respectively. To control for false positives, we adjusted probabilities by applying a false discovery rate of 0.01 [40] using the R function p.adjust. Second, we detected outliers using the program lositan [41] that also uses the fdist method, but without a hierarchical approach. We ran 50 000 simulations, the false discovery rate was set at 0.05, and used an infinite allele mutation model. Finally, we used a Bayesian approach to estimate the probability that each locus is subject to selection, using bayescan 2.1 [42]. The analysis was run with 5000 iterations and prior odds of eight, with a false discovery rate of 0.01. Based on these results, we classified each locus into one of three categories: (i) loci with *f*_ST_ values significantly higher than expected under neutrality, (ii) loci with *f*_ST_ values significantly lower than expected, and (iii) neutral loci with *f*_ST_ values within the expected range. The possible adaptive value of the outlier loci was explored by blasting their sequences in the NCBI nucleotide database and looking for match with genes and their functions. The search on the database nucleotide collection (nr/nt) was optimized for somewhat similar sequences (Blastn), with the default algorithm options.

To test for genetic structure, we conducted hierarchical AMOVAs and calculated pairwise *f*_ST_ [43] using arlequin; for the latter, sequential Bonferroni corrections were applied [44]. Discriminant analyses of principal components (DAPC) [45] were executed using adegenet [46] for R (R Development Core Team 2015). In addition, we ran the Bayesian clustering method implemented in structure [47]. To test for isolation by distance (IBD), we compared matrixes of *f*_ST_/(1 − *f*_ST_) and minimum ocean distance with Mantel tests performed using genepop [48]. For details, see electronic supplementary material, methods.

## 3. Results

A total of 1174 loci were obtained for 93 individuals. The three outlier loci methods combined identified 26 outlier loci with high *f*_ST_. The arlequin method identified 25 loci before the false discovery rate corrections and seven after the corrections. Nine loci, and one locus, were identified as outliers by lositan and bayescan, respectively. Of the nine loci identified by lositan, eight were also identified by arlequin. The outlier locus identified by bayescan was also found with lositan and arlequin. All 26 outlier loci identified were compared with GenBank entries (BLAST search) to identify potential gene functions, but no significant alignments were found (electronic supplementary material, table S1).

For the population genetic analysis with outliers, we performed analyses with 7, 9 and 25 loci (the latter being the outlier loci identified by arlequin before further corrections) and all population genetic results remained unchanged, except for some *f*_ST_ pairwise comparisons (table 1).

**Table 1.** *F*_ST_ values between populations, for neutral loci (*n* = 1117, below asterisks) and outlier loci (*n* = 25, above asterisks). Significant values (*p* < 0.05) are indicated in italics while significant values after sequential Bonferroni corrections are indicated in bolded.

	NRS	DJI	OMA	DGA	ZAN	MAY	JNO
NRS	***	**0.1522**^a^	**0.1919**	**0.2944**	**0.3139**	**0.3030**	**0.3080**
DJI	*0*.*0055*	***	**0.1825**	**0.2889**	**0.3292**	**0.3516**	**0.3533**
OMA	*0*.*0046*	**0.0075**	***	**0.3469**	**0.3399**	**0.3768**	**0.3420**
DGA	**0.0056**	**0.0088**	**0.0157**	***	*0*.*0738*	**0.0714**^b^	**0.1309**^c^
ZAN	0.0030	0.0016	**0.0060**	−0.0016	***	**0.0870**^b^	0.0273
MAY	−0.0033	**0.0050**	**0.0063**	0.0006	−0.0022	***	**0.0821**^a^
JNO	0.0008	**0.0067**	**0.0039**	*0*.*0018*	−0.0010	−0.0038	***

^a^Comparisons not significant when using the datasets with 9 and 7 outlier loci.

^b^Comparison not significant when using the dataset with 9 outliers.

^c^Comparison not significant when using the dataset with 7 outliers.

The fdist method from arlequin without false discovery rate correction that identified 25 outlier loci with high *f*_ST_ (potentially under directional selection), also identified 32 outliers potentially under balancing selection (lower than expected *f*_ST_). Based on these results, we generated three datasets for the population genetic analysis: (i) all loci (*n* = 1174), (ii) neutral loci (*n* = 1117), and (iii) outlier loci (*n* = 25). Note that the 32 outliers thought to be under balancing selection were excluded from the neutral dataset.

Analyses of all loci (*n* = 1174) showed evidence of a population structure within our study region. We found a global *F*_ST_ of 0.0127 (*p* < 0.0001). Pairwise *F*_ST_ comparisons (electronic supplementary material, table S2) indicate that DJI and OMA are significantly different from all other populations, while the NRS is different from all except MAY and JNO. An AMOVA showed a very low but significant divergence between the NWIO (Red Sea, Djibouti and Oman) and the WIO (Chagos, Zanzibar, Mayotte, Juan de Nova) (*F*_CT_ = 0.0099, *p* = 0.0225). However, structure analysis did not resolve any population clusters, while DAPC results suggest that Oman is distinct from other populations (electronic supplementary material, figure S1). Finally, there was statistically significant IBD (electronic supplementary material, figure S2), but the slope is low and there is not a strong model fit.

Analyses of the neutral dataset (*n* = 1117) indicated high genetic connectivity and weak differentiation between the two provinces (NWIO and WIO). Global *F*_ST_ was low but significant (0.0057, *p* < 0.0001), Pairwise *F*_ST_ comparisons showed that the population in Oman (OMA) stood out and it was significantly different from all other populations (table 1), while Djibouti (DJI) was significantly different from all except Zanzibar (ZAN). An AMOVA demonstrated low but significant divergence between the NWIO and the WIO provinces (*F*_CT_ = 0.0041, *p* = 0.0283, electronic supplementary material, table S3), while the DAPC analysis shows a close relationship among all populations except Oman (figure 1). structure analysis indicated *K* = 1 (figure 1). Finally, there was evidence of weak and not significant IBD (figure 1).

Analyses of the outlier loci (*n* = 25) showed strong genetic differentiation between provinces. Global *F*_ST_ for the outliers was 0.3271 (*p* < 0.0001), and all the pairwise comparisons were significant except between Zanzibar and Diego Garcia (Chagos Archipelago), and between Zanzibar and Juan de Nova (table 1). An AMOVA supports the distinction between the NWIO and WIO provinces (*F*_CT_ = 0.2349, *p* = 0.0342, electronic supplementary material, table S3). The DAPC analysis also identified separation of the NWIO and WIO provinces (figure 1), while the results from structure suggest the presence of two clusters (*K* = 2) that closely match the DAPC results (figure 1). Outliers revealed significant IBD (figure 1), but the slope is low and there is not a strong model fit.

## 4. Discussion

The present study revealed significant genetic structure between Northwestern and Western Indian Ocean populations of *D. trimaculatus,* demonstrating concordance between intraspecific phylogeographic boundaries and biogeographic boundaries [49]. In addition, Oman appears as a distinct population from all the others, and Djibouti is distinct from most populations. At least some of the divergence appears to be driven by the outlier loci. These showed a clear difference between provinces that was consistent across analyses (structure, DAPC, AMOVA), and the DAPC results for this dataset indicate that the populations of Oman, Djibouti and the Northern Red Sea are distinct from each other. These results suggest that gene flow is variable across the genome and it may be affected by different processes and/or operate at different scales. However, it is important to acknowledge that variation in recombination rate could also explain patterns of divergence across the genome [50].

Habitat discontinuities, deep-water upwellings and the direction of prevailing currents (figure 1) could be responsible for contemporary isolation between the Northwestern and Western Indian Ocean provinces. Seasonal upwelling brings cold and nutrient-rich waters to southern Oman and the Somali coast, creating large areas unsuitable for the development of coral reef habitat. In addition, currents and complex topography may divert larvae and prevent dispersal between these provinces [21,51]. If the divergences revealed in the outlier dataset are due to isolation and not adaptation, then these loci should be subject to the effects of drift and show similar patterns to the neutral loci. Our outlier dataset does, in fact, show similar—but stronger—signals compared to the neutral loci. AMOVAs based on the neutral and outlier loci demonstrate weak but significant structures between provinces (see also electronic supplementary material, table S2).

Sea-level fluctuations may also contribute to the observed pattern, as the Red Sea was subject to periods of extreme isolation when sea level dropped as much as 130 m below current levels during the Pleistocene [21]. In some cases, this repeated isolation led to speciation, while in others it only led to population differentiation, as seen here in *D. trimaculatus.* After the last glacial maximum 26.5 to 19 kya, populations of many species began to expand into the Red Sea and Persian Gulf as habitat opened up [52]. When a subset of individuals at the leading edge of a population expansion moves into a new territory, their particular alleles increase in frequency, a phenomenon called ‘allele surfing' [53]. Unlike most other demographic effects, allele surfing generally does not affect all loci, so it can impact neutral allele frequencies in ways that mimic the patterns of directional selection [53,54] and could be responsible for the results that are more evident in outlier loci.

In contrast with the more stable WIO, the NWIO is one of the most variable and environmentally extreme regions in the tropical oceans [21]. Such differences could be selecting for different traits across provinces in *D. trimaculatus* and other species. During the summer months, the waters between the Arabian Peninsula and the Red Sea become the world's hottest sea, while in the winter they become one of the coldest environments for coral reef growth [55]. The Red Sea experiences large spatio-temporal fluctuations in physical conditions and a unique north–south environmental gradient in salinity, temperature and primary productivity [21] (see electronic supplementary material, figure S3). Reefs in both the Red Sea and Gulf of Oman are known to have high variability in environmental factors such as temperature and salinity [22,56,57]. Adaptation to these highly variable environments might drive the high rates of endemism in the region [21] and may affect the survival of recruits from non-native populations. There is a possibility that the outlier loci are under selection and reflect adaptive divergence; however, this hypothesis is less likely. Isolation by distance is a neutral pattern, yet a weak trend was detected in the outlier dataset. In addition, we could not clearly identify genes involved in specific adaptations (electronic supplementary material, table S3), nor exclude the possibility of false positives.

While it is difficult to distinguish between divergence driven by selection and drift, it is important to note that these processes are not mutually exclusive and could be acting in concert on populations found around the region, given its complex geologic history and heterogeneous environment. It is possible that physical barriers between the provinces are semipermeable, allowing for restricted dispersal, and environmental contrasts between provinces reinforce those barriers through selection. Because population sizes fluctuate with sea level, the founder effect can also influence our results: population expansions after isolation can promote adaptation if colonizing individuals carry beneficial mutations [58]. In our view, multiple processes are probably at play in the study region, and carefully designed experiments are needed to disentangle their particular roles.

Despite the lack of a clear causal mechanism, our data can be used along with data on genetic connectivity of other species to identify the common genetic breaks that need to be considered for the conservation of biodiversity and evolutionary processes in the poorly studied Western Indian Ocean region. Our results suggest that the Red Sea and Arabian populations should be managed separately from the greater Western Indian Ocean population, and the role of adaptive versus neutral variation must be examined further.

## Supplementary Material

Supplementary materials and methods (extended)

Reviewer comments

## Supplementary Material

Table S1

## Supplementary Material

Table S2

## Supplementary Material

Table S3

## Supplementary Material

Figure S2

## Supplementary Material

Figure S1

## Supplementary Material

Figure S3

## Data accessibility

Data available from the Dryad Digital Repository at: https://doi.org/10.5061/dryad.bn457rr [59]. Raw fastq files are available at NCBI's SRA (accession nos. SAMN09273106-198) and the associated metadata can be found on the Genomic Observatories MetaDatabase (GeOMe: https://geome-db.org/ [60]).
