# Supplementary material for: RADseq analyses reveal concordant Indian Ocean biogeographic and phylogeographic boundaries in the reef fish Dascyllus trimaculatus
Source: R Soc Open Sci. 2019 May 29;6(5):172413. doi: 10.1098/rsos.172413 (PMC6549976; doi:10.1098/rsos.172413)
Supplement: Supplementary materials and methods (extended) [file rsos172413supp1.docx]

**SUPPLEMENTARY MATERIALS AND METHODS (extended)**

*Double-digest RADseq protocol*

The library was prepared using the double-digest RADseq protocol [1], with the following modifications. We used SphI and MluCl restriction enzymes (New England Biolabs). Pools consisted of 16 barcoded individuals. Size selection of 376-450 bp was carried out using a 2% agarose Pippin Prep**®** cassette (Sage Science). Unique Illumina Indexes were added to each pool, and the libraries were amplified using a Real-Time PCR library amplification kit (Kapa Biosystems). The concentration of each pool was quantified using a High Sensitivity Kit on a 2100 Bioanalyzer (Agilent Technologies), and then standardized and merged into a single pool. The library was sequenced on a single Illumina HiSeq 2000 lane, at the UCLA Neuroscience Genomics Core facility.

*Raw data filtering and loci assembly*

We obtained 140 million raw reads (100 bp single end). Raw data was de-multiplexed, trimmed to 95 bp and reads with Phred scores below 10 were discarded, using the “process_rad_tags” script available in stacks v1.09 [2, 3]. Sequencing resulted in 127.7 million quality filtered reads. Individuals with less than 25,000 retained reads were discarded.

Loci were assembled using the stacks de novo_map.pl pipeline. We chose a minimum of three identical reads to create a stack (m=3), three mismatches allowed between loci within an individual (M=3), five mismatches when aligning reads (N=5), and two mismatches when building the catalog (n=2). Assembly in stacks resulted in a total of 535,904 loci. We used the “populations” script to filter loci and create output files, making sure that loci were shared between the seven populations (p=7), in at least 65% of individuals within a group (r=0.65) and with coverage of 8x (m=8).

*Data analysis: Genetic structure*

AMOVAs were performed in arlequin [4] with 10,000 permutations to test for genetic structure between the NWIO and WIO provinces. Pairwise *F*_ST_ values [5] were calculated between all populations, using 10,000 permutations in arlequin. A discriminant analysis of principal components (DAPC) [6] was executed using adegenet [7] for R (R Development Core Team 2015). The DAPC plot represents the individuals as dots and the groups (populations) as inertia ellipses. Ellipse centers are at the gravity center of each population’s cloud of points. The plot uses the best number of principal components (PCs) that we identified with the cross-validation method (“xValDapc” function) on each analysis. In addition, we ran the Bayesian clustering method implemented in structure [8] with correlated allele frequencies in an admixture model, one million MCMC and 100,000 burnin chains. The most likely number of clusters (K) was determined by a combination of methods, first by looking at the assignment of the individuals. If most individuals are admixed, then we would expect no population structure and a K of 1. If there was more than one cluster, we applied the Evanno method [9] using structure harvester [10].

distruct [11] was used for the graphics in Fig. 1. To test for IBD we compared matrixes of fst/(1-fst) and minimum ocean distance, estimated by plugging the coordinates and measuring the closest ocean distances with google earth 7.1.2 (Google Inc., Mountain View, CA, USA). Mantel tests were performed with genepop [12].

**Supplementary references:**

[1] Peterson BK, Weber JN, Kay EH, Fisher HS, Hoekstra HE. 2012 Double digest RADseq: an inexpensive method for de novo SNP discovery and genotyping in model and non-model species. *PLoS ONE* **7**, e37135. (doi:10.1371/journal.pone.0037135)

[2] Catchen JM, Amores A, Hohenlohe P, Cresko W, Postlethwait JH. 2011 Stacks: building and genotyping loci de novo from short-read sequences. *G3: Genes, Genomes, Genetics* **1**, 171-182. (doi:10.1534/g3.111.000240)

[3] Catchen J, Hohenlohe PA, Bassham S, Amores A, Cresko WA. 2013 Stacks: an analysis tool set for population genomics. *Mol. Ecol.* **22**, 3124-3140. (doi:10.1111/mec.12354)

[4] Excoffier L, Lischer HE. 2010 Arlequin suite ver 3.5: a new series of programs to perform population genetics analyses under Linux and Windows. *Mol. Ecol. Resour.* **10**, 564-567. (doi:10.1111/j.1755-0998.2010.02847.x)

[5] Weir BS, Cockerham CC. 1984 Estimating F-statistics for the analysis of population structure. *Evolution* **38**, 1358-1370. (doi:10.2307/2408641)

[6] Jombart T, Devillard S, Balloux F. 2010 Discriminant analysis of principal components: a new method for the analysis of genetically structured populations. *BMC Genet.* **11**, 94. (doi:10.1186/1471-2156-11-94)

[7] Jombart T. 2008 adegenet: a R package for the multivariate analysis of genetic markers. *Bioinformatics* **24**, 1403-1405. (doi:10.1093/bioinformatics/btn129)

[8] Pritchard JK, Stephens M, Donnelly P. 2000 Inference of population structure using multilocus genotype data. *Genetics* **155**, 945-959.

[9] Evanno G, Regnaut S, Goudet J. 2005 Detecting the number of clusters of individuals using the software STRUCTURE: a simulation study. *Mol. Ecol.* **14**, 2611-2620. (doi:10.1111/j.1365-294X.2005.02553.x)

[10] Earl DA, vonHoldt BM. 2012 STRUCTURE HARVESTER: a website and program for visualizing STRUCTURE output and implementing the Evanno method. *Conserv. Genet. Resour.* **4**, 359-361. (doi:10.1007/s12686-011-9548-7)

[11] Rosenberg NA. 2004 DISTRUCT: a program for the graphical display of population structure. *Mol. Ecol. Notes* **4**, 137-138. (doi:10.1046/j.1471-8286.2003.00566.x)

[12] Raymond M, Rousset F. 1995 GENEPOP (version 1.2): population genetics software for exact tests and ecumenicism. *J. Hered.* **86**, 248-249.
