# Supplementary material for: RADseq analyses reveal concordant Indian Ocean biogeographic and phylogeographic boundaries in the reef fish Dascyllus trimaculatus
Source: R Soc Open Sci. 2019 May 29;6(5):172413. doi: 10.1098/rsos.172413 (PMC6549976; doi:10.1098/rsos.172413)
Supplement: Table S1 [file rsos172413supp2.docx]

**Table S1.** Results of a standard nucleotide BLAST search. None of the 26 outlier loci had significant alignments (Search updated Feb 3rd, 2019). We only show here results for nine loci that were identified as outliers by one of the three methods after applying a false discovery rate.

| **Locus** | **Sequence/Match** | **Query cover** | **E-value** | **Identity** |
| --- | --- | --- | --- | --- |
| 18029^1^ | *Lateolabrax maculatus* linkage group 24 sequence. Accession: CP032599.1 | 80% | 5e-13 | 84% |
| 42642 | *Lateolabrax maculatus* linkage group 24 sequence. Accession: CP032599.1 | 50% | 2e-06 | 90% |
| 14528 | *Lateolabrax maculatus* linkage group 24 sequence. Accession: CP032599.1 | 90% | 6e-12 | 81% |
| 63454 | *Helicobacter pylori* DNA, complete genome, strain: ATCC43504. Accession: AP017632.1 | 44% | 0.13 | 86% |
| 61468 | PREDICTED: *Bombus impatiens* tyrosine-protein phosphatase non-receptor type 14 (LOC100741352), transcript variant X4, mRNA. Accession: XM_012388492.2 | 25% | 0.45 | 100% |
| 24280 | *Cyprinus carpio* genome assembly common carp genome, scaffold 000000138. Accession: LN590755.1 | 63% | 7e-05 | 82% |
| 41527 | *Larimichthys crocea* genome assembly, chromosome: I. Accession: LT972175.1 | 35% | 0.037 | 91% |
| 60375^2^ | *Cottoperca gobio* genome assembly, chromosome: 2. Accession: LR131927.1 | 58% | 0.003 | 80% |
| 27426^3^ | PREDICTED: *Numida meleagris* pleckstrin homology, MyTH4 and FERM domain containing H2 (PLEKHH2), transcript variant X3, mRNA. Accession: XM_021392519.1 | 33% | 0.45 | 91% |

1: Locus identified by bayescan, arlequin and lositan

2: Locus identified by lositan, and by arlequin before applying false discovery rate

3: Locus identified only by lositan
