# Supplementary material for: RADseq analyses reveal concordant Indian Ocean biogeographic and phylogeographic boundaries in the reef fish Dascyllus trimaculatus
Source: R Soc Open Sci. 2019 May 29;6(5):172413. doi: 10.1098/rsos.172413 (PMC6549976; doi:10.1098/rsos.172413)
Supplement: Table S2 [file rsos172413supp3.docx]

**Table S2**. *F*_ST_ values between populations, for all loci n=1,174. Significant values (p<0.05) are indicated in italics, while significant values after sequential Bonferroni corrections are indicated in bolded.

|  | NRS | DJI | OMA | DGA | ZAN | MAY | JNO |
| --- | --- | --- | --- | --- | --- | --- | --- |
| NRS | **** |  |  |  |  |  |  |
| DJI | **0.0071** | **** |  |  |  |  |  |
| OMA | **0.0074** | **0.0095** | **** |  |  |  |  |
| DGA | **0.0124** | **0.0141** | **0.0231** | **** |  |  |  |
| ZAN | **0.0107** | **0.0084** | **0.0131** | -0.0013 | **** |  |  |
| MAY | *0.0042* | **0.0120** | **0.0153** | *0.0010* | -0.001 | **** |  |
| JNO | *0.0079* | **0.0136** | **0.0109** | *0.0028* | -0.002 | -0.0031 | **** |
