# Supplementary material for: RADseq analyses reveal concordant Indian Ocean biogeographic and phylogeographic boundaries in the reef fish Dascyllus trimaculatus
Source: R Soc Open Sci. 2019 May 29;6(5):172413. doi: 10.1098/rsos.172413 (PMC6549976; doi:10.1098/rsos.172413)
Supplement: Table S3 [file rsos172413supp4.docx]

**Table S3**. AMOVA combinations for neutral (n=1,117) and outlier loci (n=25).

| **Dataset** | **Groups defined** | **FCT** | **Variation explained by the groups** | **Probability** |
| --- | --- | --- | --- | --- |
| Neutral  (n=1,117) | NWIO and WIO provinces | 0.0041 | 0.41% | p=**0.0283** |
|  | Red Sea + Djibouti group, and Oman+WIO group | 0.0005 | 0.05% | p=0.4780 |
|  | Red Sea + Oman group, and Djibouti+WIO group | 0.0035 | 0.35% | p=0.0879 |
| Divergent outliers (n=25) | NWIO and WIO provinces | 0.2349 | 23 % | **p=0.0342** |
|  | Red Sea + Djibouti group, and Oman+WIO group | 0.1054 | 10.54% | p=0.1417 |
|  | Red Sea + Oman group, and Djibouti+WIO group | 0.1649 | 16.49% | P=0.1095 |
