# Supplementary figures and images for: RADseq analyses reveal concordant Indian Ocean biogeographic and phylogeographic boundaries in the reef fish Dascyllus trimaculatus
Source: R Soc Open Sci. 2019 May 29;6(5):172413. doi: 10.1098/rsos.172413 (PMC6549976; doi:10.1098/rsos.172413)

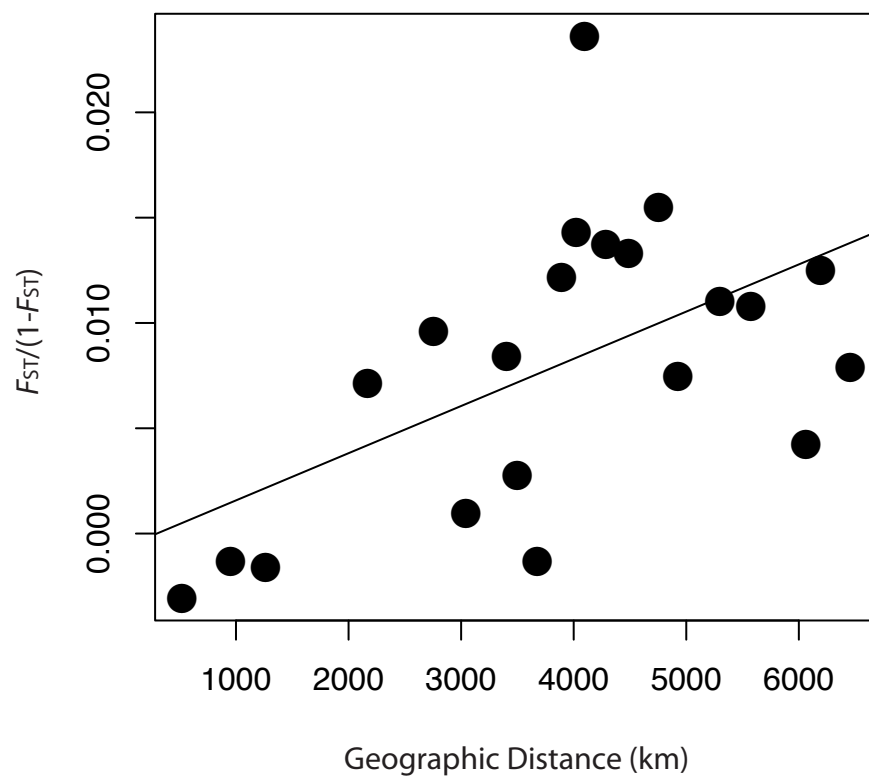

Supplement: Figure S2 [file rsos172413supp5.pdf]

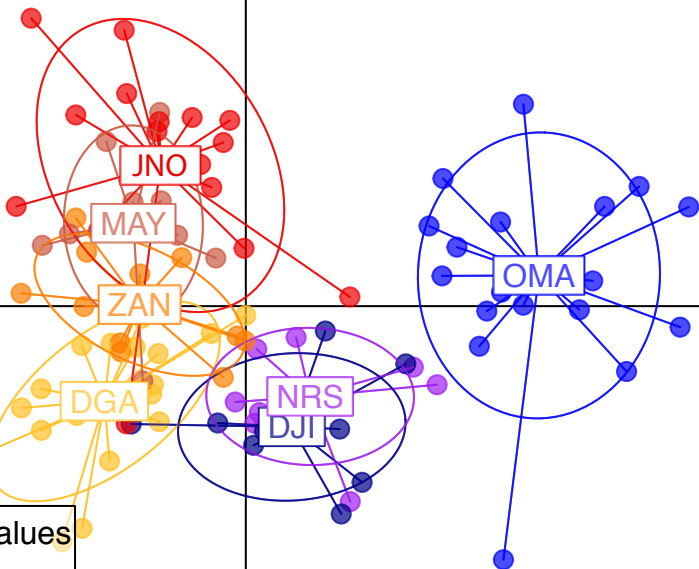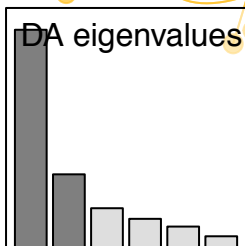

Supplement: Figure S1 [file rsos172413supp6.pdf]

Chlorophyll a concentration 4km [mg/m-3]  
Dec 2010-Dec 2014

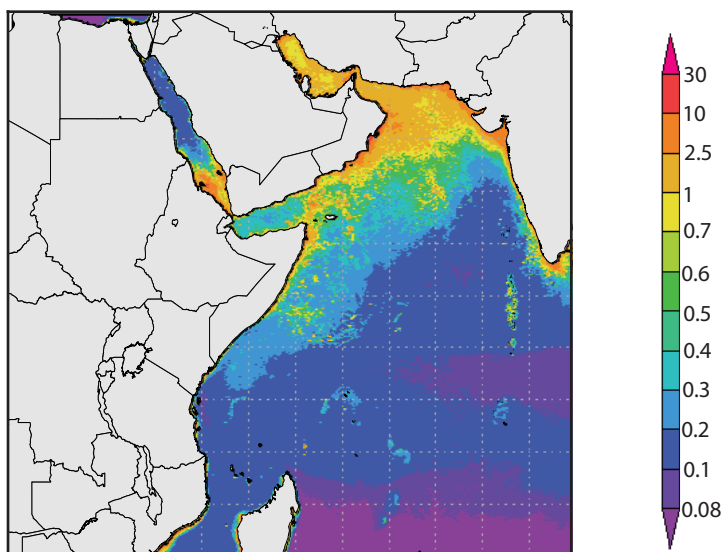

Sea surface temperature 4km [C]  
Dec 2010-Dec 2014

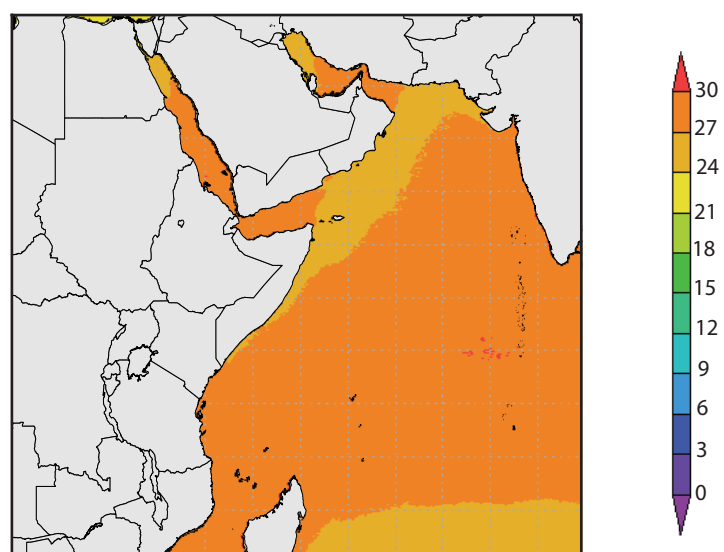

Supplement: Figure S3 [file rsos172413supp7.pdf]
